# Bis[4-(3-amino­phen­oxy)phen­yl] ketone

**DOI:** 10.1107/S1600536809025951

**Published:** 2009-07-11

**Authors:** Yang Wang, Xin-yi Zhu, Xiao-yan Ma, Guo-wei Gao, Jian Men

**Affiliations:** a College of Chemistry, Sichuan University, Chengdu 610064, People’s Republic of China; b College of Materials and Chemical Engineering, Chengdu University of Technology, Chengdu 610059, People’s Republic of China

## Abstract

In the mol­ecule of the title compound, C_25_H_20_N_2_O_3_, the dihedral angles formed by adjacent benzene rings are 66.75 (8), 48.37 (8) and 71.43 (9)°. In the crystal structure, centrosymmetrically related mol­ecules are linked into dimers by inter­molecular N—H⋯O hydrogen bonds.

## Related literature

For the properties and synthesis of the title compound, see: Wilson *et al.* (1990 ▶); Mehdipour-Ataei & Saidi (2008 ▶). For the applications of the title compound, see: Rao & Prabhakaran (1992 ▶).
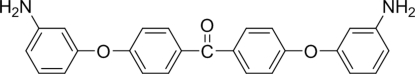


## Experimental

### 

#### Crystal data

C_25_H_20_N_2_O_3_
*M* *_r_* = 396.43
Triclinic, 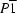

*a* = 7.370 (3) Å
*b* = 11.856 (3) Å
*c* = 12.319 (3) Å
α = 101.79 (4)°
β = 95.10 (4)°
γ = 107.86 (3)°
*V* = 989.6 (6) Å^3^
*Z* = 2
Mo *K*α radiation
μ = 0.09 mm^−1^
*T* = 292 K
0.48 × 0.42 × 0.23 mm

#### Data collection

Enraf–Nonius CAD-4 diffractometer
Absorption correction: none
3693 measured reflections
3682 independent reflections
2206 reflections with *I* > 2σ(*I*)
*R*_int_ = 0.006
3 standard reflections every 200 reflections intensity decay: 1.5%

#### Refinement

*R*[*F* ^2^ > 2σ(*F* ^2^)] = 0.053
*wR*(*F* ^2^) = 0.165
*S* = 1.05
3682 reflections
288 parameters
H atoms treated by a mixture of independent and constrained refinement
Δρ_max_ = 0.23 e Å^−3^
Δρ_min_ = −0.24 e Å^−3^

### 

Data collection: *DIFRAC* (Gabe & White, 1993 ▶); cell refinement: *DIFRAC*; data reduction: *NRCVAX* (Gabe *et al.*, 1989 ▶); program(s) used to solve structure: *SHELXS97* (Sheldrick, 2008 ▶); program(s) used to refine structure: *SHELXL97* (Sheldrick, 2008 ▶); molecular graphics: *ORTEP-3 for Windows* (Farrugia, 1997 ▶); software used to prepare material for publication: *SHELXL97*.

## Supplementary Material

Crystal structure: contains datablocks global, I. DOI: 10.1107/S1600536809025951/rz2344sup1.cif

Structure factors: contains datablocks I. DOI: 10.1107/S1600536809025951/rz2344Isup2.hkl

Additional supplementary materials:  crystallographic information; 3D view; checkCIF report

## Figures and Tables

**Table 1:** Hydrogen-bond geometry (Å, °)

*D*—H⋯*A*	*D*—H	H⋯*A*	*D*⋯*A*	*D*—H⋯*A*
N1—H1*N*1⋯O1^i^	0.92 (4)	2.32 (4)	3.223 (5)	164 (3)

Symmetry code: (i) 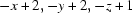.

**Fig. 1.**
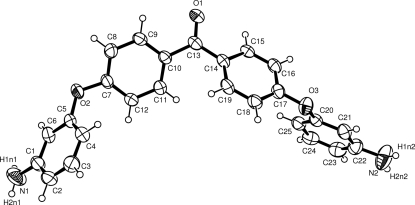
The molecular structure of the title compound, with displacement ellipsoids drawn at the 30% probability level.

## supplementary crystallographic information

### Comment

Aromatic polyimides has found useful applications in aircraft technology, space vehicles, sea transport equipment and other applications due to their excellent thermal stability, good mechanical properties, low dielectric constants and intrinsic purity (Wilson *et al.*, 1990). The title compound is an important raw material for the synthesis of aromatic polyimides, as the presence of ether and ketone groups connected by aromatic rings greatly improves the chain flexibility (Rao & Prabhakaran, 1992; Mehdipour-Ataei & Saidi, 2008). Herein, we report the synthesis and crystal structure of the title compound.

The structure of the title compound (Fig. 1) is not planar. The dihedral angle between the two central benzene rings, ring A (C7–C12) and ring B (C14–C19), is 48.37 (8)°. Ring A forms a dihedral angle of 66.75 (8)° with the C1–C6 benzene ring. The corresponding dihedral angle between ring B and the C20–C25 benzene ring is 71.43 (9)°. The plane formed by atoms C10, C14, O1 and C13, makes a dihedral angle of 22.28 (12)° and 31.23 (8)° with ring A and B, respectively. The crystal structure is stabilized by N—H···O hydrogen bonds (Table 1) linking centrosymmetrically related molecules into dimers.

### Experimental

4,4'-Difluorobenzophenone (11.0 g, 0.05 mol), *m*-aminophenol (22.0 g, 0.20 mol) and anhydrous potassium carbonate (14.0 g, 0.10 mol) were dissolved in a solution of toluene (60 ml) and *N*,*N*-dimethylformamide (100 ml) in a three-necked flask. The mixture was heated to reflux and water was removed by azeotropic distillation. After complete dehydration, the mixture was poured to a large excess of ice water. Then, the precipitated solid was collected by filtration and recrystallized from ethanol to obtain a tan solid (16.5 g, 76% yield, m.p.411–413 K). Red single crystals suitable for X-ray diffraction were obtained by slow evaporation at room temperature of a toluene solution.

### Refinement

H-atoms bound to nitrogen atoms were located in a difference Fourier map and refined isotropically. The remaining H atoms were positioned geometrically (C—H = 0.93 Å) and refined using a riding model, with *U*_iso_(H) = 1.2*U*_eq_(C).

### Crystal data

**Table:**

C_25_H_20_N_2_O_3_	*Z* = 2
*M**_r_* = 396.43	*F*(000) = 416
Triclinic, *P*1	*D*_x_ = 1.330 Mg m^−^^3^
Hall symbol: -P 1	Mo *K*α radiation, λ = 0.71073 Å
*a* = 7.370 (3) Å	Cell parameters from 23 reflections
*b* = 11.856 (3) Å	θ = 5.4–5.6°
*c* = 12.319 (3) Å	µ = 0.09 mm^−^^1^
α = 101.79 (4)°	*T* = 292 K
β = 95.10 (4)°	Block, red
γ = 107.86 (3)°	0.48 × 0.42 × 0.23 mm
*V* = 989.6 (6) Å^3^	

### Data collection

**Table:**

Enraf–Nonius CAD-4 diffractometer	*R*_int_ = 0.006
Radiation source: fine-focus sealed tube	θ_max_ = 25.5°, θ_min_ = 1.7°
graphite	*h* = −8→8
ω/2θ scans	*k* = −4→14
3693 measured reflections	*l* = −14→14
3682 independent reflections	3 standard reflections every 200 reflections
2206 reflections with *I* > 2σ(*I*)	intensity decay: 1.5%

### Refinement

**Table:**

Refinement on *F*^2^	Secondary atom site location: difference Fourier map
Least-squares matrix: full	Hydrogen site location: mixed
*R*[*F*^2^ > 2σ(*F*^2^)] = 0.053	H atoms treated by a mixture of independent and constrained refinement
*wR*(*F*^2^) = 0.165	*w* = 1/[σ^2^(*F*_o_^2^) + (0.0943*P*)^2^] where *P* = (*F*_o_^2^ + 2*F*_c_^2^)/3
*S* = 1.05	(Δ/σ)_max_ < 0.001
3682 reflections	Δρ_max_ = 0.23 e Å^−^^3^
288 parameters	Δρ_min_ = −0.24 e Å^−^^3^
0 restraints	Extinction correction: *SHELXL97* (Sheldrick, 2008), Fc^*^=kFc[1+0.001xFc^2^λ^3^/sin(2θ)]^-1/4^
Primary atom site location: structure-invariant direct methods	Extinction coefficient: 0.027 (5)

### Special details

**Table:**

Geometry. All e.s.d.'s (except the e.s.d. in the dihedral angle between two l.s. planes) are estimated using the full covariance matrix. The cell e.s.d.'s are taken into account individually in the estimation of e.s.d.'s in distances, angles and torsion angles; correlations between e.s.d.'s in cell parameters are only used when they are defined by crystal symmetry. An approximate (isotropic) treatment of cell e.s.d.'s is used for estimating e.s.d.'s involving l.s. planes.
Refinement. Refinement of *F*^2^ against ALL reflections. The weighted *R*-factor *wR* and goodness of fit *S* are based on *F*^2^, conventional *R*-factors *R* are based on *F*, with *F* set to zero for negative *F*^2^. The threshold expression of *F*^2^ > σ(*F*^2^) is used only for calculating *R*-factors(gt) *etc*. and is not relevant to the choice of reflections for refinement. *R*-factors based on *F*^2^ are statistically about twice as large as those based on *F*, and *R*- factors based on ALL data will be even larger.

### Fractional atomic coordinates and isotropic or equivalent isotropic displacement parameters (Å^2^)

**Table:**

	*x*	*y*	*z*	*U*_iso_*/*U*_eq_	
O1	0.4068 (2)	0.59493 (16)	0.42259 (15)	0.0665 (5)	
O2	1.2554 (2)	0.95837 (18)	0.55618 (15)	0.0746 (6)	
O3	0.0557 (2)	0.57862 (15)	0.86702 (15)	0.0623 (5)	
N1	1.7663 (5)	1.3024 (3)	0.7708 (4)	0.0988 (11)	
H1N1	1.729 (5)	1.346 (3)	0.725 (3)	0.116 (15)*	
H2N1	1.840 (5)	1.333 (3)	0.828 (3)	0.109 (15)*	
N2	−0.3694 (6)	0.6550 (4)	1.1145 (3)	0.1179 (13)	
H1N2	−0.378 (6)	0.581 (4)	1.131 (3)	0.119 (14)*	
H2N2	−0.399 (8)	0.712 (5)	1.153 (5)	0.19 (3)*	
C1	1.6441 (3)	1.1849 (2)	0.7619 (2)	0.0591 (7)	
C2	1.6600 (4)	1.1216 (3)	0.8431 (2)	0.0655 (7)	
H2	1.7550	1.1581	0.9064	0.079*	
C3	1.5354 (4)	1.0050 (3)	0.8303 (2)	0.0631 (7)	
H3	1.5464	0.9638	0.8859	0.076*	
C4	1.3949 (3)	0.9477 (2)	0.7371 (2)	0.0544 (6)	
H4	1.3115	0.8685	0.7286	0.065*	
C5	1.3819 (3)	1.0112 (2)	0.6574 (2)	0.0509 (6)	
C6	1.5030 (3)	1.1280 (2)	0.6682 (2)	0.0558 (7)	
H6	1.4901	1.1689	0.6126	0.067*	
C7	1.0692 (3)	0.8848 (2)	0.5542 (2)	0.0518 (6)	
C8	0.9903 (3)	0.7910 (2)	0.4600 (2)	0.0549 (6)	
H8	1.0622	0.7789	0.4031	0.066*	
C9	0.8035 (3)	0.7150 (2)	0.4507 (2)	0.0517 (6)	
H9	0.7494	0.6512	0.3869	0.062*	
C10	0.6938 (3)	0.73176 (19)	0.53531 (19)	0.0450 (6)	
C11	0.7750 (3)	0.8300 (2)	0.6275 (2)	0.0511 (6)	
H11	0.7023	0.8444	0.6834	0.061*	
C12	0.9628 (3)	0.9070 (2)	0.6377 (2)	0.0543 (6)	
H12	1.0165	0.9728	0.7000	0.065*	
C13	0.4904 (3)	0.6501 (2)	0.5182 (2)	0.0505 (6)	
C14	0.3844 (3)	0.63592 (19)	0.6137 (2)	0.0451 (6)	
C15	0.1849 (3)	0.61103 (19)	0.5934 (2)	0.0475 (6)	
H15	0.1256	0.6057	0.5218	0.057*	
C16	0.0742 (3)	0.5943 (2)	0.6765 (2)	0.0521 (6)	
H16	−0.0585	0.5779	0.6615	0.063*	
C17	0.1629 (3)	0.6021 (2)	0.7822 (2)	0.0502 (6)	
C18	0.3584 (3)	0.6242 (2)	0.8042 (2)	0.0552 (6)	
H18	0.4165	0.6282	0.8757	0.066*	
C19	0.4680 (3)	0.6404 (2)	0.7205 (2)	0.0539 (6)	
H19	0.6000	0.6546	0.7356	0.065*	
C20	−0.0391 (3)	0.6582 (2)	0.9107 (2)	0.0470 (6)	
C21	−0.1542 (3)	0.6200 (2)	0.9866 (2)	0.0560 (6)	
H21	−0.1692	0.5443	1.0020	0.067*	
C22	−0.2484 (4)	0.6946 (3)	1.0405 (2)	0.0665 (7)	
C23	−0.2239 (4)	0.8067 (3)	1.0155 (3)	0.0705 (8)	
H23	−0.2847	0.8584	1.0514	0.085*	
C24	−0.1107 (4)	0.8412 (2)	0.9382 (2)	0.0635 (7)	
H24	−0.0982	0.9157	0.9210	0.076*	
C25	−0.0142 (3)	0.7687 (2)	0.8849 (2)	0.0550 (6)	
H25	0.0647	0.7937	0.8334	0.066*	

### Atomic displacement parameters (Å^2^)

**Table:**

	*U*^11^	*U*^22^	*U*^33^	*U*^12^	*U*^13^	*U*^23^
O1	0.0462 (10)	0.0733 (12)	0.0579 (12)	0.0017 (9)	−0.0019 (9)	0.0004 (9)
O2	0.0424 (10)	0.1008 (14)	0.0539 (12)	−0.0116 (9)	0.0015 (8)	0.0184 (10)
O3	0.0645 (11)	0.0627 (10)	0.0744 (13)	0.0286 (9)	0.0262 (10)	0.0307 (9)
N1	0.088 (2)	0.0664 (18)	0.105 (3)	−0.0097 (16)	−0.007 (2)	0.0029 (18)
N2	0.136 (3)	0.121 (3)	0.142 (3)	0.069 (3)	0.092 (3)	0.059 (3)
C1	0.0427 (13)	0.0545 (15)	0.0680 (18)	0.0079 (12)	0.0060 (13)	0.0026 (13)
C2	0.0490 (15)	0.0799 (19)	0.0562 (17)	0.0168 (14)	−0.0056 (13)	0.0050 (14)
C3	0.0568 (16)	0.0775 (18)	0.0614 (18)	0.0285 (14)	0.0051 (14)	0.0232 (15)
C4	0.0461 (13)	0.0527 (14)	0.0602 (16)	0.0117 (11)	0.0058 (12)	0.0129 (12)
C5	0.0317 (11)	0.0637 (15)	0.0497 (15)	0.0084 (11)	0.0058 (11)	0.0092 (12)
C6	0.0421 (13)	0.0581 (15)	0.0662 (17)	0.0109 (12)	0.0091 (12)	0.0217 (13)
C7	0.0373 (12)	0.0609 (15)	0.0496 (15)	0.0040 (11)	0.0012 (11)	0.0184 (12)
C8	0.0405 (13)	0.0699 (16)	0.0527 (16)	0.0158 (12)	0.0086 (11)	0.0151 (13)
C9	0.0449 (13)	0.0532 (14)	0.0467 (15)	0.0110 (11)	−0.0030 (11)	0.0025 (11)
C10	0.0368 (12)	0.0481 (13)	0.0460 (14)	0.0111 (10)	−0.0010 (10)	0.0101 (11)
C11	0.0395 (13)	0.0554 (14)	0.0544 (16)	0.0122 (11)	0.0072 (11)	0.0107 (12)
C12	0.0446 (14)	0.0606 (15)	0.0450 (14)	0.0052 (11)	−0.0002 (11)	0.0075 (11)
C13	0.0394 (13)	0.0462 (13)	0.0580 (17)	0.0100 (11)	−0.0019 (12)	0.0062 (12)
C14	0.0324 (11)	0.0424 (12)	0.0548 (15)	0.0075 (9)	−0.0002 (10)	0.0100 (11)
C15	0.0379 (12)	0.0471 (13)	0.0491 (14)	0.0094 (10)	−0.0064 (11)	0.0066 (11)
C16	0.0331 (12)	0.0540 (14)	0.0647 (17)	0.0119 (10)	0.0018 (12)	0.0109 (12)
C17	0.0476 (14)	0.0433 (12)	0.0589 (16)	0.0128 (10)	0.0094 (12)	0.0140 (11)
C18	0.0460 (14)	0.0611 (15)	0.0527 (16)	0.0105 (11)	−0.0043 (12)	0.0176 (12)
C19	0.0336 (12)	0.0553 (14)	0.0661 (17)	0.0072 (10)	−0.0026 (12)	0.0167 (12)
C20	0.0384 (12)	0.0478 (13)	0.0482 (14)	0.0095 (10)	−0.0022 (11)	0.0089 (11)
C21	0.0472 (13)	0.0560 (15)	0.0651 (17)	0.0156 (12)	0.0063 (12)	0.0189 (13)
C22	0.0605 (17)	0.0778 (19)	0.0654 (19)	0.0269 (15)	0.0164 (14)	0.0179 (15)
C23	0.0716 (19)	0.0689 (18)	0.072 (2)	0.0342 (15)	0.0058 (16)	0.0045 (15)
C24	0.0725 (18)	0.0523 (15)	0.0602 (18)	0.0216 (14)	−0.0049 (14)	0.0076 (13)
C25	0.0520 (14)	0.0529 (14)	0.0546 (16)	0.0118 (12)	0.0022 (12)	0.0128 (12)

### Geometric parameters (Å, °)

**Table:**

O1—C13	1.226 (3)	C10—C11	1.384 (3)
O2—C7	1.377 (3)	C10—C13	1.484 (3)
O2—C5	1.390 (3)	C11—C12	1.383 (3)
O3—C17	1.387 (3)	C11—H11	0.9300
O3—C20	1.390 (3)	C12—H12	0.9300
N1—C1	1.384 (4)	C13—C14	1.478 (3)
N1—H1N1	0.92 (4)	C14—C19	1.386 (3)
N1—H2N1	0.79 (4)	C14—C15	1.396 (3)
N2—C22	1.383 (4)	C15—C16	1.373 (3)
N2—H1N2	0.93 (4)	C15—H15	0.9300
N2—H2N2	0.84 (5)	C16—C17	1.377 (3)
C1—C6	1.381 (4)	C16—H16	0.9300
C1—C2	1.384 (4)	C17—C18	1.374 (3)
C2—C3	1.373 (4)	C18—C19	1.374 (3)
C2—H2	0.9300	C18—H18	0.9300
C3—C4	1.375 (4)	C19—H19	0.9300
C3—H3	0.9300	C20—C21	1.370 (3)
C4—C5	1.368 (3)	C20—C25	1.373 (3)
C4—H4	0.9300	C21—C22	1.387 (4)
C5—C6	1.370 (3)	C21—H21	0.9300
C6—H6	0.9300	C22—C23	1.387 (4)
C7—C8	1.371 (3)	C23—C24	1.364 (4)
C7—C12	1.379 (3)	C23—H23	0.9300
C8—C9	1.374 (3)	C24—C25	1.380 (4)
C8—H8	0.9300	C24—H24	0.9300
C9—C10	1.394 (3)	C25—H25	0.9300
C9—H9	0.9300		
			
C7—O2—C5	120.22 (19)	C7—C12—H12	120.5
C17—O3—C20	119.63 (18)	C11—C12—H12	120.5
C1—N1—H1N1	117 (2)	O1—C13—C14	119.2 (2)
C1—N1—H2N1	115 (3)	O1—C13—C10	119.3 (2)
H1N1—N1—H2N1	124 (4)	C14—C13—C10	121.5 (2)
C22—N2—H1N2	118 (2)	C19—C14—C15	117.8 (2)
C22—N2—H2N2	112 (4)	C19—C14—C13	124.5 (2)
H1N2—N2—H2N2	127 (5)	C15—C14—C13	117.6 (2)
C6—C1—N1	119.0 (3)	C16—C15—C14	121.7 (2)
C6—C1—C2	118.8 (2)	C16—C15—H15	119.2
N1—C1—C2	122.2 (3)	C14—C15—H15	119.2
C3—C2—C1	120.1 (3)	C15—C16—C17	118.9 (2)
C3—C2—H2	120.0	C15—C16—H16	120.6
C1—C2—H2	120.0	C17—C16—H16	120.6
C2—C3—C4	121.5 (3)	C18—C17—C16	120.8 (2)
C2—C3—H3	119.2	C18—C17—O3	118.1 (2)
C4—C3—H3	119.2	C16—C17—O3	121.0 (2)
C5—C4—C3	117.7 (2)	C19—C18—C17	119.9 (2)
C5—C4—H4	121.2	C19—C18—H18	120.0
C3—C4—H4	121.2	C17—C18—H18	120.0
C4—C5—C6	122.2 (2)	C18—C19—C14	120.9 (2)
C4—C5—O2	122.2 (2)	C18—C19—H19	119.6
C6—C5—O2	115.4 (2)	C14—C19—H19	119.6
C5—C6—C1	119.8 (2)	C21—C20—C25	122.1 (2)
C5—C6—H6	120.1	C21—C20—O3	114.4 (2)
C1—C6—H6	120.1	C25—C20—O3	123.5 (2)
C8—C7—O2	115.7 (2)	C20—C21—C22	119.7 (2)
C8—C7—C12	121.3 (2)	C20—C21—H21	120.1
O2—C7—C12	122.9 (2)	C22—C21—H21	120.1
C7—C8—C9	119.2 (2)	N2—C22—C21	120.0 (3)
C7—C8—H8	120.4	N2—C22—C23	121.2 (3)
C9—C8—H8	120.4	C21—C22—C23	118.8 (3)
C8—C9—C10	121.1 (2)	C24—C23—C22	120.0 (3)
C8—C9—H9	119.4	C24—C23—H23	120.0
C10—C9—H9	119.4	C22—C23—H23	120.0
C11—C10—C9	118.4 (2)	C23—C24—C25	121.9 (2)
C11—C10—C13	122.8 (2)	C23—C24—H24	119.0
C9—C10—C13	118.6 (2)	C25—C24—H24	119.0
C12—C11—C10	120.9 (2)	C20—C25—C24	117.4 (2)
C12—C11—H11	119.5	C20—C25—H25	121.3
C10—C11—H11	119.5	C24—C25—H25	121.3
C7—C12—C11	119.0 (2)		
			
C6—C1—C2—C3	−0.7 (4)	O1—C13—C14—C19	147.9 (2)
N1—C1—C2—C3	−179.9 (3)	C10—C13—C14—C19	−33.6 (3)
C1—C2—C3—C4	0.9 (4)	O1—C13—C14—C15	−29.1 (3)
C2—C3—C4—C5	−0.5 (4)	C10—C13—C14—C15	149.3 (2)
C3—C4—C5—C6	−0.1 (4)	C19—C14—C15—C16	1.5 (3)
C3—C4—C5—O2	174.8 (2)	C13—C14—C15—C16	178.8 (2)
C7—O2—C5—C4	43.0 (3)	C14—C15—C16—C17	−0.1 (3)
C7—O2—C5—C6	−141.8 (2)	C15—C16—C17—C18	−1.1 (3)
C4—C5—C6—C1	0.2 (4)	C15—C16—C17—O3	−176.1 (2)
O2—C5—C6—C1	−174.9 (2)	C20—O3—C17—C18	116.9 (2)
N1—C1—C6—C5	179.4 (3)	C20—O3—C17—C16	−68.0 (3)
C2—C1—C6—C5	0.1 (4)	C16—C17—C18—C19	0.9 (4)
C5—O2—C7—C8	−147.3 (2)	O3—C17—C18—C19	176.0 (2)
C5—O2—C7—C12	36.4 (4)	C17—C18—C19—C14	0.6 (4)
O2—C7—C8—C9	−178.7 (2)	C15—C14—C19—C18	−1.8 (3)
C12—C7—C8—C9	−2.4 (4)	C13—C14—C19—C18	−178.8 (2)
C7—C8—C9—C10	0.0 (4)	C17—O3—C20—C21	174.4 (2)
C8—C9—C10—C11	2.4 (3)	C17—O3—C20—C25	−8.5 (3)
C8—C9—C10—C13	177.4 (2)	C25—C20—C21—C22	−0.5 (4)
C9—C10—C11—C12	−2.4 (3)	O3—C20—C21—C22	176.6 (2)
C13—C10—C11—C12	−177.1 (2)	C20—C21—C22—N2	178.0 (3)
C8—C7—C12—C11	2.4 (4)	C20—C21—C22—C23	0.3 (4)
O2—C7—C12—C11	178.4 (2)	N2—C22—C23—C24	−177.0 (3)
C10—C11—C12—C7	0.1 (4)	C21—C22—C23—C24	0.8 (4)
C11—C10—C13—O1	154.3 (2)	C22—C23—C24—C25	−1.6 (4)
C9—C10—C13—O1	−20.4 (3)	C21—C20—C25—C24	−0.2 (4)
C11—C10—C13—C14	−24.2 (3)	O3—C20—C25—C24	−177.1 (2)
C9—C10—C13—C14	161.1 (2)	C23—C24—C25—C20	1.3 (4)

### Hydrogen-bond geometry (Å, °)

**Table:**

*D*—H···*A*	*D*—H	H···*A*	*D*···*A*	*D*—H···*A*
N1—H1N1···O1^i^	0.92 (4)	2.32 (4)	3.223 (5)	164 (3)

Symmetry codes: (i) −*x*+2, −*y*+2, −*z*+1.

## References

[bb1] Farrugia, L. J. (1997). *J. Appl. Cryst.* **30**, 565.

[bb2] Gabe, E. J., Le Page, Y., Charland, J.-P., Lee, F. L. & White, P. S. (1989). *J. Appl. Cryst.* **22**, 384–387.

[bb3] Gabe, E. J. & White, P. S. (1993). Am. Crystallogr. Assoc. Pittsburgh Meet. Abstract PA104.

[bb4] Mehdipour-Ataei, S. & Saidi, S. (2008). *Polym. Adv. Technol.* **19**, 889-894.

[bb5] Rao, V. L. & Prabhakaran, P. V. (1992). *Eur. Polym. J.* **28**, 363–366.

[bb6] Sheldrick, G. M. (2008). *Acta Cryst.* A**64**, 112–122. 10.1107/S0108767307043930 18156677

[bb7] Wilson, D., Stengenberger, H. D. & Hergenrother, P. M. (1990). In *Polyimides* New York: Chapman and Hall.

